# 3D Scene Reconstruction Using Omnidirectional Vision and LiDAR: A Hybrid Approach

**DOI:** 10.3390/s16111923

**Published:** 2016-11-16

**Authors:** Michiel Vlaminck, Hiep Luong, Werner Goeman, Wilfried Philips

**Affiliations:** 1 Department of Telecommunications and Information Processing, Ghent University, Sint-Pietersnieuwstraat 41, iMinds, Ghent 9000, Belgium; hiep.luong@telin.ugent.be (H.L.); philips@telin.ugent.be (W.P.); 2 Sweco/Grontmij, Ghent 9000, Belgium; werner.goeman@grontmij.be

**Keywords:** 3D point cloud registration, Iterative Closest Point (ICP), LiDAR scanning, loop closure, surface reconstruction, Velodyne, Ladybug

## Abstract

In this paper, we propose a novel approach to obtain accurate 3D reconstructions of large-scale environments by means of a mobile acquisition platform. The system incorporates a Velodyne LiDAR scanner, as well as a Point Grey Ladybug panoramic camera system. It was designed with genericity in mind, and hence, it does not make any assumption about the scene or about the sensor set-up. The main novelty of this work is that the proposed LiDAR mapping approach deals explicitly with the inhomogeneous density of point clouds produced by LiDAR scanners. To this end, we keep track of a global 3D map of the environment, which is continuously improved and refined by means of a surface reconstruction technique. Moreover, we perform surface analysis on consecutive generated point clouds in order to assure a perfect alignment with the global 3D map. In order to cope with drift, the system incorporates loop closure by determining the pose error and propagating it back in the pose graph. Our algorithm was exhaustively tested on data captured at a conference building, a university campus and an industrial site of a chemical company. Experiments demonstrate that it is capable of generating highly accurate 3D maps in very challenging environments. We can state that the average distance of corresponding point pairs between the ground truth and estimated point cloud approximates one centimeter for an area covering approximately 4000 m${}^{2}$. To prove the genericity of the system, it was tested on the well-known Kitti vision benchmark. The results show that our approach competes with state of the art methods without making any additional assumptions.

## 1. Introduction

Reconstructing the 3D scene using a mobile observer, also referred to as mobile mapping, is used in a variety of applications ranging from navigational tasks for autonomous vehicles to facility condition assessment or cartography. The former application imposes a real-time constraint on the processing time of the used algorithms, whereas the latter allows the system to take more computation time. The application that we have in mind aims at construction site monitoring. The goal there is to obtain a 3D model of the environment in a reasonable amount of time, which spans the duration of the intervention. To this end, we propose a system that combines both a LiDAR sensor (Velodyne HDL32-e) and a panoramic camera system (Ladybug 3) to perceive the environment. Some systems exist that rely on the output of a regular camera to obtain 3D reconstructions [1,2], known in the literature as (visual) structure from motion (SfM). In the case of sequential (or incremental) SfM, the problem is also referred to as simultaneous localization and mapping (SLAM). However, visual SfM produces rather sparse 3D reconstructions, which require much post-processing to make them more dense. Moreover, they are prone to drift and tend to fail when the images are overexposed because of the abundance of sunlight. For this reason, the core of our mobile mapping system is based on the output of a LiDAR sensor, aided by images of a regular camera to perform loop detection. Often times, mobile mapping systems are also relying on GPS to estimate the trajectory of the observer. However, in the environments we have in mind, i.e., construction sites with many overhanging or protruding structures (e.g., pipelines), the GPS signal is too weak. In closed indoor environments, it is even completely lacking. Therefore, we developed a system that is fully GPS-independent. In summary, we propose an entire 3D reconstruction system that covers both the odometry and mapping problem. It incorporates the alignment of consecutive point clouds, the fusion with a global 3D map and a loop closure mechanism to cope with drift. The main goal of our approach is to make the system as generic as possible and, hence, to make as little assumptions as possible about the environment and about the sensor set-up. Doing so, we are able to use our system in combination with a drone-mounted LiDAR sensor and camera in the future. The main contribution of this work is that our solution deals explicitly with the inhomogeneous density of point clouds produced by LiDAR scanners. This latter refers to the fact that some parts of the point cloud have a higher point density than others due to the inclination of the laser beams and the proximity of the objects present in the scene. Generally, regions close to the sensor origin will be densely sampled, whereas more distant objects will be sparsely sampled. As has been shown in numerous studies [3,4,5], traditional point-to-point registration techniques (e.g., Iterative Closest Point (ICP)) fail in these situations. For that reason, we propose a number of improvements over standard point-to-point strategies and incorporate them in one framework. The main contributions can be summarized as follows:

1. We propose a surface analysis technique that is used to build a topological space on top of the point cloud, providing for each point its ideal neighborhood and taking into account the underlying surface.
2. We keep track of a global 3D map that is continuously updated by means of a surface reconstruction technique that allows us to resample the point cloud. This way, the global 3D map is continuously improved, i.e., the noise is reduced, and the alignment of the following point clouds can be conducted more accurately.
3. The topological space is used to compute low-level features, which will be incorporated in an adapted version of the ICP algorithm to make this latter more robust. The alignment of consecutive local point clouds, as well as the alignment of a local point cloud with the global 3D map will be conducted using this improved ICP algorithm.
4. We incorporate the residual of the ICP process in the loop closure process. Based on this residual, we can predict the share of each pose estimation in the final pose error when a loop has been detected.

## 2. Related Work

Regarding LiDAR odometry and mapping, existing solutions can roughly be divided in four main classes: based on ICP, normal distribution transform (NDT), features and planar surfaces. The majority of the approaches presented in the literature are based on the iterative closest point (ICP) algorithm in order to perform scan matching and to identify the transformation between sensor poses [6,7,8]. However, the standard ICP algorithm has many limitations, and therefore, many improvements have been presented in literature. One of the main drawbacks lies in the fact that it is often difficult to find good point to point correspondences between two point clouds, especially in the case of a sparse point density or when the speed of the moving platform is too high. The authors of velocity ICP [9] therefore propose to perform velocity estimation over the ICP iterations. Doing so, the distortion of a scan due to motion can be compensated by re-projecting all points acquired during one sweep to the position of the sensor at the beginning of the sweep. In [10], the authors tackle the correspondence problem by defining low-level features that are used to guide the ICP process. The authors focus on pair-wise registration of datasets that do not exhibit large changes. They do not investigate a full sequential 3D reconstruction approach that incorporates a global 3D map or loop closure. The authors of Velodyne SLAM [11], on the other hand, incrementally build a map of the environment by aligning each newly arrived Velodyne scan with the whole map using ICP. Their global map is improved over time by adapting incoming measurements according to already existing adapted neighbors. However, the refinement of the global map is limited as they do not perform a surface analysis or re-sampling of the point cloud. Moreover, they do not incorporate a loop closure mechanism to cope with drift. Finally, some studies focus on combining the ICP-based scan matching with (stereo) visual odometry. For example, in [12], the authors propose a solution that uses the output of visual odometry to obtain a rough estimate of the ego motion upon which consecutive point clouds are registered. The generalized ICP algorithm [13] is then used to refine the motion estimation, and finally, the output is combined with reduced IMU outputs. Although these latter solutions seem effective, these studies, in contrast to ours, do not investigate how LiDAR odometry itself can be improved.

Aside from ICP, the normal distribution transform (NDT) [14] is another common technique that is often used for laser scan matching [15,16,17]. It is based on an alternative representation of a point cloud similar to an occupancy grid. In each 3D cell, a normal distribution is stored, and both scans are matched using Newton’s method. Compared to ICP-based methods, the main advantage of this method is that there is no need for correspondence estimation. The main limitation is however that the accuracy of NDT is strongly related to the cell size, which is difficult to define in the case of inhomogeneous point clouds.

Another way to cope with correspondence estimation is to introduce the use of features. Feature-based methods have significant advantages as they concentrate on strong cues, such as corners and lines and filter out irregular points, such as vegetation or tree leaves. One very interesting feature-based system has been developed by Zhang et al. and described in [18,19]. Their technique is based on the extraction of only two different shape features, representing sharp edges and planar surface patches. Edge points of the source cloud are associated with edge lines, while planar patches are matched with other planar patches in the target cloud. In [19], the same authors elaborated on their method by incorporating data from a regular camera to perform visual odometry.

Finally, in [20,21,22], the authors use yet another approach based on the registration of planar surfaces. These planes can lead to very accurate alignments as they serve as strong cues and can be estimated by an accumulation of data, hence reducing the noise. However, although these methods are very effective, the lack of planes in the scene can cause them to fail and, hence, make them less generic.

In this work, we will adopt a hybrid approach in the sense that we combine ICP-based registration with feature extraction and surface reconstruction. To this end, we adopt the strategy of [10] by incorporating low-level features that describe the underlying surface in a simple way, i.e., using three main classes, linear, planar and volumetric. We exploit these properties to identify for each point an ideal neighborhood and, hence, a topological space representing the captured scene. The low-level features will further be used to guide the ICP process in such a way that the alignment concentrates itself near the most reliable regions, i.e., lines and corners instead of irregular points or clutter. Our approach can benefit from large planar surfaces while at the same time remaining generic. It is thus more widely applicable. In addition, we keep track of a global map, which will continuously be improved and refined with newly-captured points. More specifically, we use a surface reconstruction technique after which we resample the point cloud and improve its topological space. Finally, we incorporate loop closure to cope with the remaining drift.

## 3. System

### 3.1. Acquisition Platform

**Figure 1:**
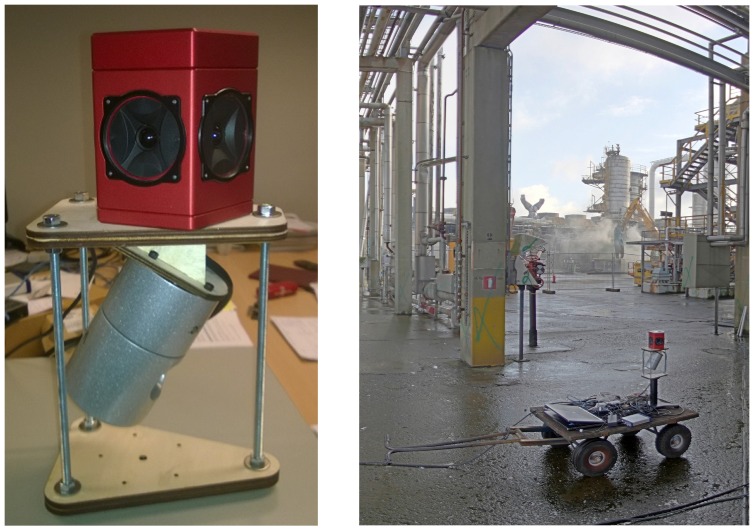
Two pictures of our mobile acquisition platform Vellady mounted on a kitchen cart (**right**) and close-up (**left**). The platform consists of a Ladybug panoramic camera (mounted at the top) and a Velodyne HDL32-e LiDAR scanner (mounted at the bottom).

**Figure 2:**
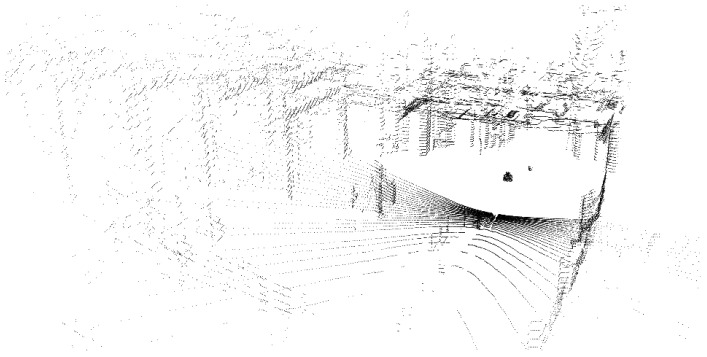
A 360${}^{\circ }$ point cloud acquired by the Velodyne HDL-32e LiDAR scanner, associated with the stitched lady bug image of Figure 3, captured at the Dow chemical company.

**Figure 3:**
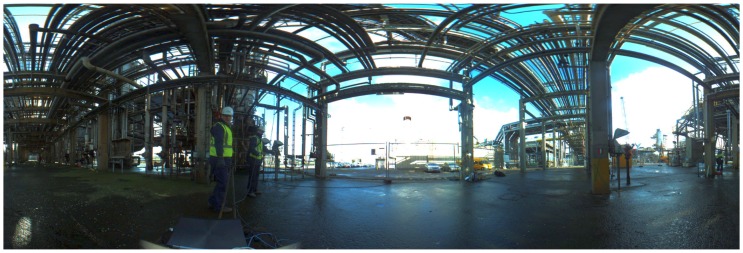
An image acquired by stitching the six images of the Ladybug together. The image shows the starting point of a video sequence captured at the Dow chemical company.

Our acquisition platform consists of a Velodyne High Definition LiDAR (HDL-32e) scanner combined with a Ladybug panoramic camera system. Because of this combination, we named it the Vellady platform. As can be seen in Figure 1, the Ladybug camera is mounted perpendicular to the ground plane, whereas the Velodyne is tilted on its head, making an angle of approximately 66${}^{\circ }$ with the ground plane. The Velodyne LiDAR scanner is equipped with 32 lasers mounted collinear and covering a vertical FOV of 41.3${}^{\circ }$, hence resulting in a vertical resolution of 1.29${}^{\circ }$. The vertical FOV covers 30.67${}^{\circ }$ below the middle point of the Velodyne and 10.67${}^{\circ }$ above it. The head is continuously spinning at approximately 10 Hz, resulting in a horizontal FOV of 360${}^{\circ }$. Figure 2 shows an example of a point cloud obtained by this Velodyne sensor. The Ladybug on the other hand is a fixed system that comes in the form of a pentagonal prism consisting of five vertical-oriented sides, each one incorporating a camera. A sixth camera is mounted on top pointing upwards. It is shooting images at one frame per second. A stitched image obtained by this Ladybug camera system, corresponding with the point cloud of Figure 2, is depicted in Figure 3. We mounted this Vellady platform on a moving vehicle, i.e., a kitchen cart or four-wheel trolley (see Figure 1). Our platform is hence not able to rotate around its roll angle. A rotation about its pitch angle is only possible in the case that the ground plane has a slope, but these situations were not encountered during the experiments. However, we did not exploit these facts as we want to retain the option to mount the platform on a drone in the future. Our system thus operates as a full six degrees of freedom SLAM algorithm incorporating three unknown position coordinates *x*, *y*, *z* and three rotation angles $\theta_{x}$, $\theta_{y}$, $\theta_{z}$. The main features of our platform are summarized in Table 1.

### 3.2. Terminology

As mentioned in the previous section, the Velodyne is spinning its head, thereby producing 360${}^{\circ }$ point clouds. A full rotation of the head is also referred to as a sweep. Throughout this paper, we use right subscript *k*, $k\in Z^{+}$ to indicate the sweep number and $P_{k}$ to indicate the point cloud perceived during sweep *k*. This point cloud is expressed using the local coordinate system of the Velodyne $V_{k}$ at the time that sweep *k* has started. We will use right superscript *i* to denote a single point $p_{k}^{i}$. Finally, we define $I_{j}$ to denote the *j*-th set of images. Recall that the Ladybug only outputs six images simultaneously each second, and hence, some point clouds will have no accompanying visual information. However, both sensors were synchronized, and as a result, the following condition is always warranted: $\forall \;j\in Z^{+},\exists \;k\in Z^{+}:t\left(I_{j}\right)=t\left(P_{k}\right)$. In this expression, *t* is a function that returns the timestamp at which respectively the *j*-th set of images was captured and sweep *k* was started. As the Velodyne is spinning at a frequency of 10 Hz, there will only be visual information available for every tenth point cloud $P_{k}$.

### 3.3. Reference Coordinate System

**Figure 4:**
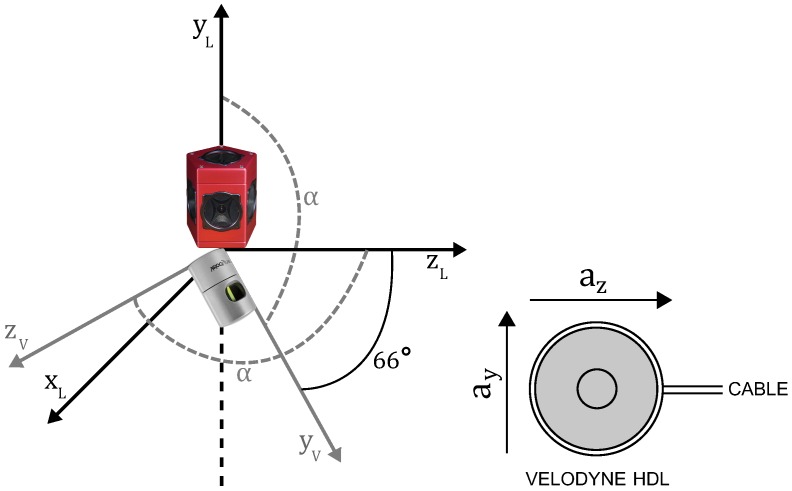
Schematic drawing of the rotation performed after nullifying the offset of the sensor origins. The angle about the x-axis, i.e., the pitch angle, was computed using the output of the two-axis accelerometer $a=(a_{y},a_{z})$ from the Velodyne HDL. In this figure, the subscript L denotes the coordinate system of the Ladybug, whereas V denotes the coordinate system of the Velodyne.

As both sensor outputs $P_{k}$ and $I_{k}$ are expressed in their own coordinate system, we need to define a reference coordinate system to harmonize both. To ease this task, the platform was designed in a way that the origin of both sensors is collinear. Since the Ladybug was put perpendicular to the ground plane, it is sufficient to determine the orientation of the Velodyne sensor with respect to the ground plane and nullify the offset of the origins of both sensors. As the Velodyne scanner incorporates three gyroscopes and three associated two-axis accelerometers, the transformation can be derived from one of its accelerometers. Specifically, given the accelerometer output $a=(a_{y},a_{z})$ (cf. Figure 4), the angle about the x-axis, i.e., the pitch angle, can be computed as $\alpha =\mathrm{atan}(a_{z}/a_{y})$. The final transformation $T^{\left(vel2lady\right)}\in R^{4\times 4}$ from the Velodyne coordinate system V to the Ladybug coordinate system L is then given by Equation (1):

(1) $$T^{\left(vel2lady\right)}=\left[\begin{array}{cc} R & t\\ 0_{3}^{⊤} & 1 \end{array}\right]=\left[\begin{array}{cccc} 1 & 0 & 0 & t_{x}\\ 0 & \cos\alpha & \sin\alpha & t_{y}\\ 0 & -\sin\alpha & \cos\alpha & t_{z}\\ 0 & 0 & 0 & 1 \end{array}\right].$$

Herein, the values of $t=(t_{x},t_{y},t_{z})$ were determined by subtracting the two vectors that project both origins to the ground plane. In the case of the Velodyne coordinate system V, the projection of the origin is performed after applying R. Next, the coordinate system W denotes the global coordinate system in which the final 3D point cloud is expressed. We decided to set the coordinate system of the Ladybug at the start of the first sweep $L_{0}$ as the global world coordinate system W.

### 3.4. Problem Statement

The problem can be formulated as finding the trajectory, i.e., the sequence of sensor poses $S=\{S_{1},\ldots ,S_{k},\ldots ,S_{N}\}$, that transforms each point cloud $P_{k}$ in the world coordinate system W. The poses $S_{k}$ are defined as in Equation (2):

(2) $$\begin{array}{cc} S_{k} & =\left[\begin{array}{cc} R_{k} & t_{k}\\ 0_{3}^{⊤} & 1 \end{array}\right]=T_{k,k-1}S_{k-1}, \end{array}$$

(3) $$\begin{array}{cc} S_{0} & =\left[\begin{array}{cc} R_{0} & t_{0}\\ 0_{3}^{⊤} & 1 \end{array}\right]=T^{\left(\mathrm{vel}2\mathrm{lady}\right)}. \end{array}$$

**Figure 5:**
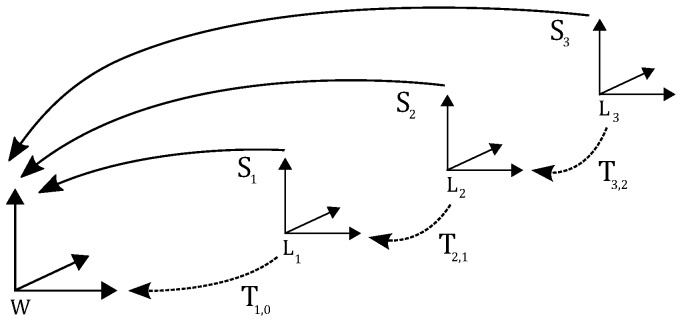
Overview of the problem statement. Each time a new point cloud $P_{k}$ has arrived, its sensor pose $S_{k}$ in relation to the world reference coordinate system $W\;=\;L_{0}$ is determined using the concatenation of the previous pose $S_{k-1}$ and the transformation $T_{k,k-1}$.

Herein, $T_{k,k-1}$ denotes the transformation of the point clouds $P_{k}$ and $P_{k-1}$ acquired in two consecutive sweeps. The reconstructed 3D point cloud $W_{k}$ after *k* sweeps have been processed is given by $W_{k}=\left\{P_{0},\ldots ,S_{k}P_{k}\right\}$. The reconstructed point cloud of the entire sequence containing *N* sweeps is finally given by $W=\{P_{0},\ldots ,S_{k}P_{k},\ldots ,S_{N}P_{N}\}$. Figure 5 depicts a schematic drawing of this problem statement.

## 4. Approach

**Figure 6:**
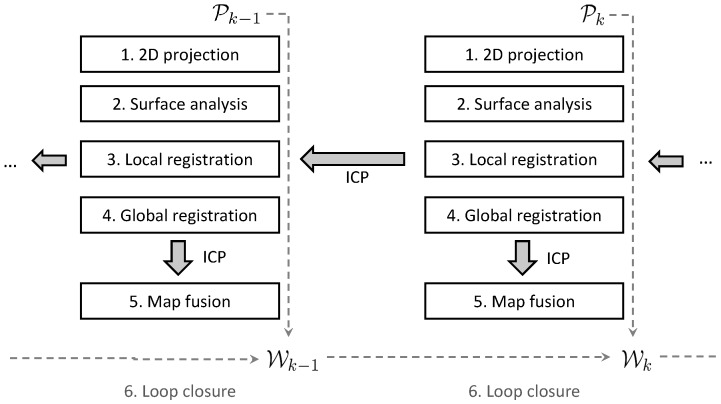
Overview of the system, which is implemented as an incremental process. Every time a consecutive point cloud $P_{k}$ has arrived, it is first projected on a 2D grid (Step 1). Next, it is analyzed to obtain some low-level surface features (Step 2), after which it is aligned (registered) with the point cloud $P_{k-1}$ acquired during the previous sweep k−1 (Step 3). The obtained transformation serves as an initial guess to subsequently align $P_{k}$ with the current world model $W_{k-1}$ (Step 4) and to fuse them together to form $W_{k}$ (Step 5). This fusion consists of re-sampling the point cloud by means of a surface reconstruction technique. Optionally, when a loop has been detected, loop closure is performed to cope with the drift and to preserve global consistency (Step 6).

A schematic overview of our approach is depicted in Figure 6. As can be seen, it is implemented as a sequential, i.e., incremental, process in which every newly-arrived point cloud $P_{k}$ is first processed and subsequently added to the current world model $W_{k}$. The process repeated for every point cloud is conducted in five steps. First, we project the generated point clouds on a 2D grid (Step 1). Subsequently, we conduct a surface analysis (Step 2), after which we perform pairwise alignment of two consecutive point clouds (Step 3), which serves as an initial guess for the current pose. Next, we register the aligned point cloud with a global 3D map (Step 4) and fuse the new points with this 3D map (Step 5). This fusion consists of re-sampling the point cloud by means of a surface reconstruction technique. Optionally, when a loop has been detected, we adopt loop closure to preserve global consistency (Step 6). This is done by means of pose graph optimization, which propagates the estimated error back in the pose graph. In the following sections, we will further clarify each of these steps.

### 4.1. 2D Projection

**Figure 7:**
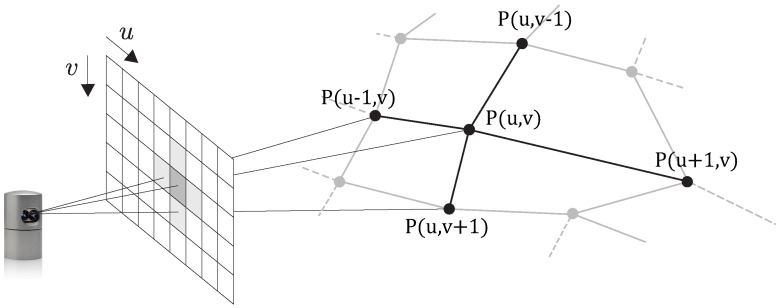
The 3D points generated by the Velodyne scanner are projected onto a 2D spherical grid. This way, we can exploit the adjacency in the 2D domain, e.g., to quickly find neighboring points in 3D.

**Figure 8:**
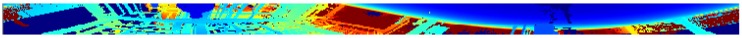
Example of a 360${}^{\circ }$ range image obtained by projecting the point cloud of Figure 2 onto a 2D grid. The blue color means that points are close-by, whereas the red color denotes that points are located further away.

The 3D points captured by the Velodyne are organized since the 32 lasers are placed collinear in the vertical direction. Because of that, we can project the 3D points onto a two-dimensional spherical grid; cf. Figure 7. Figure 8 shows an example of a 360${}^{\circ }$ range image corresponding to the point cloud of Figure 2. Using this 2D projection, we can exploit the adjacency in the pixel domain, e.g., to quickly find neighboring points in 3D. We will exploit this knowledge to perform a surface analysis as explained in Section 4.2.

### 4.2. Surface Analysis

**Figure 9:**
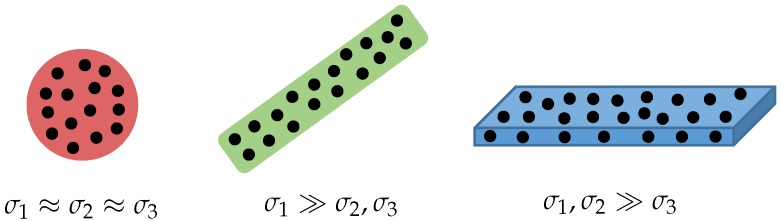
Visual representation of the low-level surface features. The values $\sigma_{i}=\sqrt{\lambda_{i}}$ represent the standard deviation along the eigenvectors. When $\sigma_{1}\approx \sigma_{2}\approx \sigma_{3}$, the points are volumetric, often times representing a scatter, such as vegetation. When $\sigma_{1}\gg \sigma_{2},\sigma_{3}$, the respective points are lying on a line between planar surfaces or are part of thin structures, such as pipelines. Finally, when $\sigma_{1},\sigma_{2}\gg \sigma_{3}$, the points are lying on a planar surface.

The surface analysis step is performed twice each iteration, once on the point cloud $P_{k}$ perceived during sweep *k* and once on the point cloud map $W_{k-1}$ after the fusion of the previous point cloud $P_{k-1}$. Our proposed algorithm is an extension of the one presented in [10]. It aims to find for each 3D point the optimal neighborhood size or the most suitable local point set that describes the underlying geometry. This is an interdependence problem, as geometrical features largely depend on the choice of the neighborhood, whereas a good neighborhood definition should rely on the local geometry and, thus, on geometrical features. Let us first define a neighborhood function *n* as $n^{r}\left(p\right):\left(x,y,z\right)\mapsto {\left(x,y,z\right)}^{N}$. In this equation, *r* denotes the search radius for neighboring points, and *N* denotes the number of points belonging to the neighborhood. Using this neighborhood, we determine the principal components of the covariance matrix of the points belonging to it. These principal components are used to decide whether the underlying surface is linear (1D), planar (2D) or volumetric (3D), cfr. Figure 9. Hence, we first compute the three eigenvalues $\lambda_{1},\lambda_{2}$ and $\lambda_{3}$ and their corresponding eigenvectors $v_{1},v_{2}$ and $v_{3}$. Next, we define the standard deviation along an eigenvector as $\sigma_{i}=\sqrt{\lambda_{i}}$ for i∈1,2,3. Using these three values, we can obtain a measure on how linear, planar or scattered the underlying surface is. To this end, we define the three dimensionality values as follows:

(4) $$\begin{array}{c} \psi_{1}=\frac{\sigma_{1}-\sigma_{2}}{\sigma_{1}},\;\psi_{2}=\frac{\sigma_{2}-\sigma_{3}}{\sigma_{1}},\;\psi_{3}=\frac{\sigma_{3}}{\sigma_{1}}. \end{array}$$

These three values are normalized such that $\psi_{1}+\psi_{2}+\psi_{3}=1$; hence, they represent a partition of unity Ψ. Finally, the dimensionality label *l* of each point is given by Equation (5):

(5) $$\begin{array}{c} l=\underset{i\in [1,3]}{\mathrm{argmax}}\left(\psi_{i}\right). \end{array}$$

When $\sigma_{1}\gg \sigma_{2},\sigma_{3}$, then $\psi_{1}$ will be larger than the two other features. This corresponds to lines between planar surfaces or thin structures, such as pipelines. On the other hand, when $\sigma_{1},\sigma_{2}\gg \sigma_{3}$, then $\psi_{2}$ will be larger, corresponding to planar surfaces. Finally, when $\sigma_{1}\approx \sigma_{2}\approx \sigma_{3}$, then $\psi_{3}$ will be larger often times representing a scatter, such as bushes or tree leaves. To determine the optimal radius $r^{*}$, we use the concept of Shannon entropy. To this end, we compute the geometrical features for a growing radius size $r\in [r_{min},r_{max}]$. The Shannon entropy for the partition $\Psi^{r}={\left\{\psi_{1},\psi_{2},\psi_{3}\right\}}_{r}$ is given by $E\left(\Psi^{r}\right)=-\psi_{1}\ln\left(\psi_{1}\right)-\psi_{2}\ln\left(\psi_{2}\right)-\psi_{3}\ln\left(\psi_{3}\right)$. This value gives a measure for the uncertainty about the dimensionality label. The optimal neighborhood radius $r^{*}$ is then defined as the minimum of the entropy function *E*:

(6) $$\begin{array}{c} r^{*}=\underset{r\in [r_{min},r_{max}]}{\mathrm{argmin}}E\left(\Psi^{r}\right). \end{array}$$

This radius $r^{*}$ leads to an initial guess of the optimal neighborhood $V_{p}^{*}$ of a point p. As points within the optimal radius $r^{*}$ can still belong to different surfaces and can have different dimensionality labels, we define a similarity measure *S* denoting the homogeneity within the neighborhood of each point:

(7) $$\begin{array}{c} S\left(n^{r}\left(p\right)\right)=\frac{1}{N}\sum_{p_{i}\in n^{r}\left(p\right)}1_{l\left(p\right)=l\left(p_{i}\right)}. \end{array}$$

In this equation, 1 represents the indicator function, and *N* is the cardinality of $n^{r}\left(p\right)$. If this value *S* is smaller than 0.5, we reconsider the optimal radius $r^{*}$ using Equation (8):

(8) $$\begin{array}{c} r^{*}=\underset{r\in [r_{min},r_{max}]}{\mathrm{argmax}}S\left(n^{r}\left(p\right)\right). \end{array}$$

In the other case, we remove the points belonging to the neighborhood of p that have another dimensionality label. Hence, we define the optimal neighborhood $V_{p}^{*}$ of a point p as in Equation (9):

(9) $$V_{p}^{*}=\left\{p_{i}:p_{i}\in n^{r^{*}}\left(p\right)\wedge l\left(p\right)=l\left(p_{i}\right)\right\}.$$

In summary, the proposed method tries to find the ideal topological space $T^{*}$ representing the underlying surface of the point cloud. For each node (or point) in $T^{*}$, we store a 19-dimensional feature vector, which is the combination of the eigen values $\lambda =\{\lambda_{1},\lambda_{2},\lambda_{3}\}$, the eigen vectors $V=\{v_{1}$, $v_{2},v_{3}\}$, the dimensionality values $\Psi =\{\psi_{1},\psi_{2},\psi_{3}\}$, the dimensionality label *l* and the values $r^{*}$ and $E^{*}$. The last feature we define represents the omnivariance and is given by $O=\prod_{i\in [1,3]}\sigma_{i}$. One of the most important features in this vector is the local surface normal $n_{i}$ of a point $p_{i}$, which can be approximated by the eigenvector $v_{3}$ corresponding to the smallest eigenvalue $\lambda_{3}$ of the principal components, which was computed using the optimal neighborhood.

### 4.3. Local Registration

**Figure 10:**
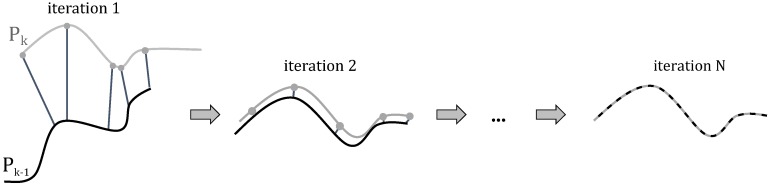
Graphical representation of the ICP algorithm. In every iteration, four main steps are conducted: (1) point selection; (2) correspondence estimation; (3) weighting; and (4) transformation estimation. In every iteration, the transformation is updated until convergence has been reached and the two scans are perfectly aligned.

The majority of the solutions presented in the literature to find the alignment of consecutive scans or point clouds are based on the iterative closest point (ICP) algorithm. As the name suggests, it is an iterative method that consists of four main steps in each iteration: (1) point selection; (2) correspondence estimation; (3) weighting; and (4) transformation estimation. In every iteration, the transformation is updated until convergence has been reached. In Figure 10, a graphical representation of the algorithm is depicted. However, the standard ICP approach has some severe limitations. One of the main issues originates from the fact that the point clouds generated by the Velodyne scanner have a sparse and inhomogeneous density. As a result, it is hard to find good point correspondences between two consecutive point clouds (referred to as source and target point cloud), as most of the time, the determined corresponding pairs will not represent the same physical point in space. This leads to point clouds that are not aligned accurately, even after a large number of iterations and, hence, to wrongly estimated sensor poses. In addition, the convergence process will be very slow. Motivated by this shortcoming, we propose to incorporate the low-level features that we derived in the surface analysis step to guide the ICP process. Doing so, we will not use the closest points in 3D as target points directly as is done in traditional ICP. Instead, we present an approach in which we use the local surface normals that were computed using the optimal neighborhood (cf. Section 4.1). We then minimize the distance from each source point to the tangent plane of its corresponding surface patch in the target point cloud. In the following, we will briefly discuss the four main sub-parts of the process.

(1) Point Selection

The goal of the point selection step is to focus on the most reliable areas for accurate registration. An area is considered as unreliable if it is located near the border between several objects or surfaces or if it belongs to a geometrically complex object. As the points corresponding with a volumetric label (l=3) are mainly corresponding to scattered points, e.g., bushes or tree leaves, we will exclude them from the estimation process, as we consider them less reliable. This is mainly due to the fact that it is hard to estimate accurate surface normals for these points, as they represent complex structures and are too sparsely sampled. In addition, we will exclude planar points for which the uncertainty, i.e., the entropy, is too high. In the experiments, the threshold for the entropy was set to 0.8.

(2) Correspondence Estimation

In contrast with existing systems, we will select the corresponding target point $p_{k-1}^{j}$ as the point having the most similar neighborhood as the source point $p_{k}^{i}$ in terms of the geometry of the underlying surface. To this end, we compute the distance from the source point to the target point in feature space. However, as it is too infeasible to compare every point in the target point cloud with the source point, we first determine corresponding candidates by selecting the closest points in Euclidean space using the 2D projection of both point clouds. More specifically, we will look for the points that are only a few pixels away in the 2D domain. Finally, the target point that has the smallest distance in feature space is chosen as the corresponding point.

(3) Weighting

In order to make the procedure more robust against correspondence outliers computed in the previous section, we incorporate robust M-estimators in the iteration process. Thus, instead of using fixed weights, we adapt them through the iterations resulting in an iteratively reweighted least squares (IRLS) ICP optimization. Compared to traditional solutions, this weighting is carried out using the distance of the corresponding pairs in feature space instead of Euclidean 3D space. This is important as the point clouds are inhomogeneous, and the distance between correct correspondence pairs, i.e., representing the same physical point, will vary greatly based on the distance of the points to the origin of the sensor. We want to emphasize that the feature vector of a point itself is not changing during consecutive ICP iterations, but its corresponding point can be chosen differently, as the matching process also depends on their Euclidean distance (cf. Section 4.3). Thus, the distance in feature space can be varying during the iterations.

(4) Transformation Estimation

In traditional ICP approaches, the transformation between point clouds is estimated by minimizing the sum of the Euclidean distances between corresponding points, known as the point-to-point distance. In our approach, we will not match points from the source point cloud $P_{k}$ with points in the target point cloud $P_{k-1}$, but rather match points of $P_{k}$ with surface patches of $P_{k-1}$. The goal is to minimize the distance between the points in $P_{k}$ with the tangent plane of the corresponding surface patch in $P_{k-1}$, known as the point-to-plane distance and given by the following error metric:

(10) $$E\left(P_{k},P_{k-1};T_{k,k-1}\right)=\sum_{i=1}^{N}w^{i}{\left(\left(T_{k,k-1}p_{k}^{i}-p_{k-1}^{c(i)}\right)\cdot n_{k-1}^{c(i)}\right)}^{2}.$$

Herein, $T_{k,k-1}$ is the estimated transformation matrix; $P_{k}$ is the source point cloud; $P_{k-1}$ is the target point cloud; $n_{k-1}^{i}$ is the surface normal according to target point; $p_{k-1}^{i}$, $w^{i}$ is the weight vector; and c is the vector containing the indices of the *N* corresponding points. Equation (11) gives the expression to derive the final transformation matrix $T_{k,k-1}$:

(11) $$T_{k,k-1}=\left[\begin{array}{cc} R_{k,k-1} & t_{k,k-1}\\ 0_{3}^{⊤} & 1 \end{array}\right]=\underset{T_{k,k-1}}{\mathrm{argmin}}E\left(P_{k},P_{k-1};T_{k,k-1}\right).$$

In order to solve this optimization problem, we adopt the method proposed by Low et al. in [23]. In that paper, a method is derived to approximate the nonlinear optimization problem with a linear least squares one in the case that the relative orientation between the two input point clouds is small. Since we consider point clouds acquired in two consecutive sweeps, this assumption is guaranteed in our case.

### 4.4. Global Registration

Once we have found the initial transformation $T_{k,k-1}$ between point clouds $P_{k}$ and $P_{k-1}$, the next step consists of fusing $P_{k}$ with the current registered point cloud $W_{k-1}$. To this end, we first transform $P_{k}$ in the world coordinate system using the estimate of the current pose, which is given by ${\hat{S}}_{k}=T_{k,k-1}S_{k-1}$ resulting in ${\hat{P}}_{k}={\hat{S}}_{k}P_{k}$. As the point cloud ${\hat{P}}_{k}$ is still not accurately aligned with $W_{k-1}$, we perform a second registration step based on ICP as explained in the previous section. However, regarding the transformation estimation, the cost function that we want to minimize is now given by Equation (12):

(12) $$T_{k,w}=\left[\begin{array}{cc} R_{k,w} & t_{k,w}\\ 0_{3}^{⊤} & 1 \end{array}\right]=\underset{T_{k,w}}{\mathrm{argmin}}E\left({\hat{P}}_{k},W_{k-1};T_{k,w}\right).$$

To find the correspondence points of ${\hat{P}}_{k}$ in $W_{k-1}$, we can still use the criterion explained in Section 4.3. However, as the world map is growing, it is practically infeasible to look in the entire map for correspondences. Fortunately, this is not necessary as corresponding points in the map will be located close to the newly-added point cloud ${\hat{P}}_{k}$, as this latter is already transformed with an estimate of the current pose ${\hat{S}}_{k}$. Therefore, we can first filter the map by means of frustum culling using the oriented bounding box of the current sweep as a box filter extended with a small offset. The principal axes of this oriented bounding box are already known as these are previously determined by the accelerometer output. This box filtering will lower the processing speed without harming the guarantee to find good point correspondences. As we cannot use the organized structure of the separated point clouds any more after they have been fused with the point cloud map, we first create a kd-tree of the points remaining after the box filtering. After the registration of ${\hat{P}}_{k}$ with $W_{k-1}$, we define $S_{k}=T_{k,w}{\hat{S}}_{k}$.

### 4.5. Map Fusion

After adding $P_{k}$ to the former world model $W_{k-1}$, the point density in this world model will increase. We can now refine the ideal topological space $T^{*}$ and hence the local surface normals in the global 3D map by re-estimating them using their new neighborhood. However, as this point cloud contains noise and has an inhomogeneous point density, we will resample it using the moving least squares (MLS) surface reconstruction algorithm [24]. In summary, this method will try to locally approximate the underlying surface by higher order polynomial interpolations between surrounding data points. Using these polynomials one can resample the point cloud and obtain more accurate estimates for the surface normals. The procedure can be described as follows. Using the ideal topological space $T^{*}$, we have for each point p an optimal neighborhood $V_{p}^{*}$, as well as the tangent plane to p defined as $H_{p}≜\left[n,d\right]$. For all points lying within this neighborhood, we can compute the distance to this tangent plane. Subsequently, we fit a polynomial in the set of distances from these points to the surface. To this end, we define a local approximation of degree *m* by a polynomial $\tilde{p}\in \Pi_{m}$ minimizing, among all $p\in \Pi_{m}$, the weighted least-squares error of Equation (13):

(13) $$\sum_{i\in I}{\left(p\left(x_{i}\right)-f_{i}\right)}^{2}\theta (||p_{i}-p||).$$

In this equation, I is the vector of indices representing the points in $V_{p}^{*}$; ${\left\{x_{i}\right\}}_{i\in I}$ are the orthogonal projections of the points ${\left\{p_{i}\right\}}_{i\in I}$ onto $H_{p}$; and $f_{i}≜\langle p_{i},n\rangle -d$ is the distance of $p_{i}$ to the tangent plane $H_{p}$. Finally, $\theta \left(x\right)=e^{-{\left(\frac{x}{\sigma_{r}}\right)}^{2}}$ represents the weighting function that is based on the distances to the tangent plane and the average separation $\sigma_{r}$ of the 3D points.

Once the parameters of the polynomials are known, we finally project the data points back on the moving least squares surface. This procedure will hence manipulate the sampled data points in such a way that they represent the underlying surface in a better way. In addition, we upsample the point cloud using voxel grid dilation in order to fill small gaps. This latter procedure will first dilate a voxel grid representation of the world model built using a predefined voxel size. After that, the resulting new points are projected to the MLS surface of the closest point in the world point cloud. With time, $W_{k-1}$ is becoming larger and larger, and as a result, the search for nearest neighbors becomes quite intractable. For that reason, we store the map as an octree data structure. Using this octree, we can work on a downsampled version of the point cloud map $W_{k-1}$. The main benefit of this octree representation is that we can keep all points, but at the same time, we can freely choose the level of density we want to use.

### 4.6. Loop Closure

Detecting loops using the acquired point clouds would be too cumbersome, as it would take ages to compare every newly-generated point cloud with the entire 3D map. For this reason, we perform loop detection on the images of the Ladybug. We adopt the same strategy presented in [25] (DBOW2) and improved by [1] (ORB-SLAM). More specifically, we adopt a bag of words (BOW) implemented as a hierarchical tree that uses a visual vocabulary. This vocabulary is built offline using ORB descriptors and converts an image into a sparse numerical vector. Each image is thus converted into a bag of word vector that is used to compare it with other images and to measure the similarity. This latter is conducted in the same way as described in [25]. In addition, an index is maintained that stores for each word in the vocabulary the list of images where it is present. Doing so, we are able to perform comparisons only against images that have some word in common with the query image.

**Figure 11:**
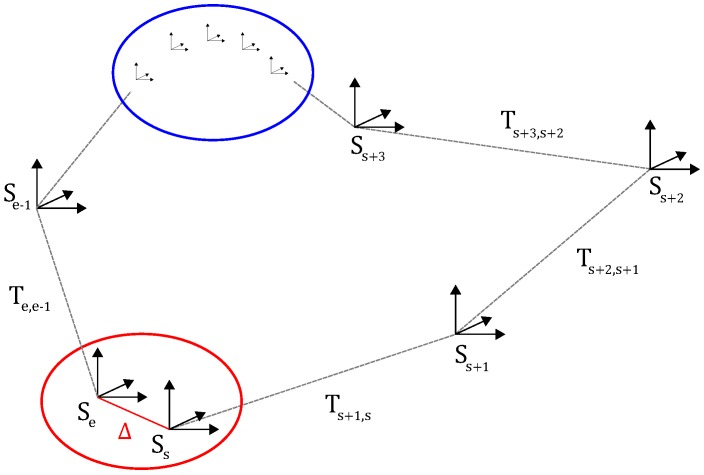
Overview of the loop closure procedure. When a loop has been detected, its loop transform Δ is considered as an error and is propagated back in the pose graph. To this end, we use the residual of the minimization step (cf. Section 4.3) to assign a weight to each link in the pose graph. We assign a higher weight for those transformations that had a high residual in the previous minimization step. The idea is that a high residual indicates that two consecutive point clouds were potentially inaccurately aligned.

Let us assume that a loop has been detected between image set $I_{j}$ and $I_{i}$. We now consider all point clouds $P_{l}$ captured in the interval $[t\left(I_{j}\right),t\left(I_{j+1}\right)[$ (recall that there are ten sweeps in the interval) and try to find the most similar point cloud $P_{k}$ captured in the interval $[t\left(I_{i}\right),t\left(I_{i+1}\right)[$. As the sensor could have visited the same location from different directions, it can happen that both ends of the loop have different orientations. Therefore, we first determine the matrix $R_{k,l}$ that describes the rotation between $P_{l}$ and $P_{k}$. It is important to note that we compute the transformation on the local point clouds, which are expressed in their own coordinate system. The rotation estimation is a two-step process, consisting of an initial rough alignment and a refinement step using the ICP algorithm. The two point clouds $P_{l}$ and $P_{k}$ that have the lowest residual after the ICP step are considered the most similar point clouds and are denoted by $P_{e}$ and $P_{s}$, representing both ends of the loop. Their poses are respectively $S_{e}$ and $S_{s}$. Next, the difference in pose, i.e., the loop transform, is given by $\Delta ={\left(R_{s,e}S_{e}\right)}^{-1}S_{s}$. This loop transform is considered as an error as both poses $R_{s,e}S_{e}$ and $S_{s}$ should be equal. The next step consists of propagating the error Δ back in the pose graph. To this end, we use the residual of the minimization step (cf. Section 4.3) to assign a weight $c_{i,j}$ to each link in the pose graph. We assign a higher weight for those transformations that had a high residual in the previous minimization step. The idea is that a high residual indicates that two consecutive point clouds were potentially inaccurately aligned. On the other hand, the ICP process will have already been converging in the right direction. Next, we define the distance between two poses $S_{k}$ and $S_{l}$ as $d\left(S_{k},S_{l}\right)=\sum_{i,j}c_{i,j}$. Herein, {i,j} denotes the set of all edges in the path from $S_{k}$ to $S_{l}$. Finally, we define a weight $w_{i}=\frac{d(S_{s},S_{i})}{d(S_{s},S_{e})}$ for each pose in the graph that specifies the fraction of the matrix Δ by which the pose has to be transformed. The poses $S_{k}$ are than updated replacing $t_{k}$ by $t_{k}w_{k}\Delta$ and $R_{k}$ by $slerp(R_{k},w_{k}\Delta )$. In this latter assignment, *slerp* denotes the spherical linear interpolation function as described in [26]. A graphical representation of this loop closure procedure is depicted in Figure 11.

## 5. Evaluation

To evaluate the system, we considered two different datasets. The first dataset we have captured ourselves. It covers different environments, including a university campus and an industrial site. The latter was captured at a chemical site of the Dow company in Terneuzen in the Netherlands. In order to be able to compare our system with the state of the art, we also performed some experiments on the well-known Kitti vision benchmark that was presented in [27].

### 5.1. Our Dataset

**Figure 12:**
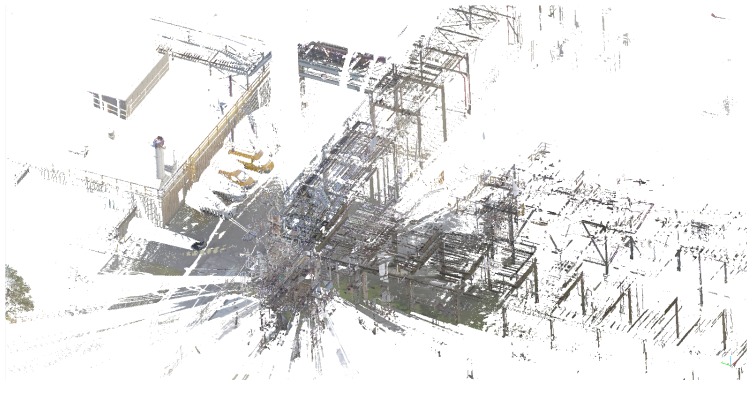
Part of the ground truth 3D point cloud of the Dow chemical site acquired using a Leica static terrestrial LiDAR scanning system.

**Figure 13:**
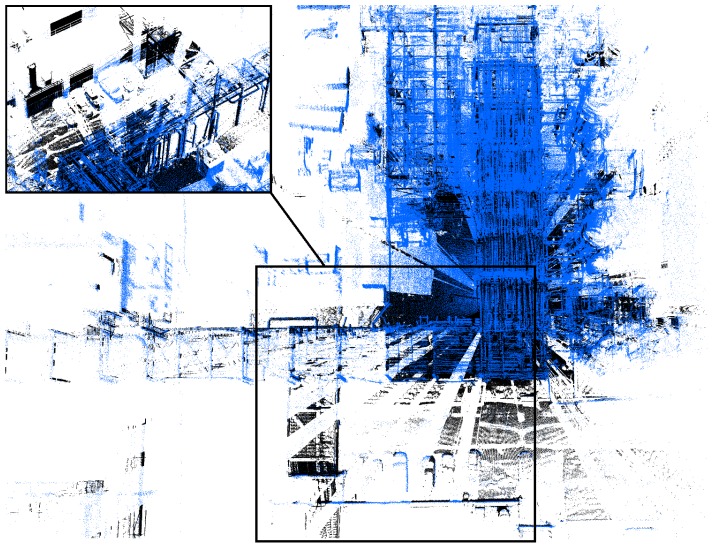
Bird’s-eye view of the ground truth (black) and reconstructed (blue) point cloud aligned with each other. Visually, one can see that the walls and edges of both point clouds are overlapping. This is best visible in areas for which the ground truth point cloud has a LiDAR shadow due to occlusion. The average distance of the corresponding dominant planes (cf. Table 2) is approximately 1 cm, whereas the average deviation in angle is $0.84^{\circ }$.

**Figure 14:**
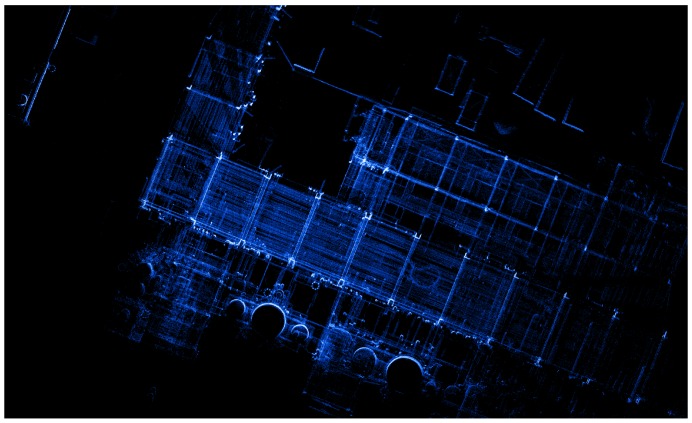
2D projection on the ground plane of both the reconstructed and the ground truth 3D model in order to evaluate the accuracy qualitatively. To obtain the image, the ground plane was removed from both point clouds, and a quantization in 2D cells was conducted. Brighter areas denote a higher point density in the cell and, hence, emphasize the overlap of both point clouds. Visually, one can notice little to no discrepancies as the walls and edges of both models are overlapping.

The first set of sequences was captured at a chemical site of the Dow company in Terneuzen. This environment is part of a disused area that is planned to be demolished. It consists of many pipelines that were formerly used to carry liquids or gases, as can be seen in Figure 3. Although this environment seems outdoors, the GPS signal is far too unreliable due to the abundance of pipelines. The acquired data consists of video sequences recorded with our Vellady platform. The speed of the platform approximated walking speed, i.e., 4 km/h. Furthermore, accurate ground truth information by means of terrestrial laser scanning was captured using a Leica system.(www.leica-geosystems.be) An image of this ground truth point cloud is depicted in Figure 12. As we do not have ground truth of the actual trajectory, we have to compare the reconstructed point cloud with the ground truth point cloud. For this comparison, it is necessary that both point clouds are aligned. Hence, we will first perform a rough alignment based on key points that we manually selected from both point clouds. Subsequently, we perform ICP to align them in a fine way. The final residual of the ICP process, i.e., the Euclidean distance between all closest point pairs, can be considered as a measure for the accuracy of the reconstruction. The experiments demonstrated that the final residual of this ICP process is approximately 2.1 cm for our reconstruction. However, as the closest point pairs do not necessarily represent the same physical points in space, the ICP residual is not a perfect measure for the evaluation of the accuracy. For that reason, we conducted another experiment in which we use the dominant planes in the scene to make the comparison. More specifically, in both point clouds, we estimate the parameters of the most dominant planes after which we determine the corresponding planes. Subsequently, we compute for all inliers of the planes from the source point cloud the distance to its corresponding plane in the target point cloud. We hence define the average distance of two corresponding planes $H_{s}≜\left[n_{s},d_{s}\right]$ and $H_{t}≜\left[n_{t},d_{t}\right]$ as $d\left(H_{s},H_{t}\right)≜\frac{1}{|H_{t}|}\sum_{p_{t}\in H_{t}}\langle p_{t},n_{s}\rangle -d_{s}$. In addition, we also define the difference in angle of the two corresponding planes as $\phi \left(H_{s},H_{t}\right)≜\mathrm{acos}\left(n_{s}\cdot n_{t}\right)$. The results are summarized in Table 2 for the eight most dominant planes in the scene. The table shows that on average, the deviation in angle of two corresponding planes is approximately $0.84^{\circ }$, whereas the average distance between two planes is approximately 1 cm. In Figure 13, an image is depicted in which both the ground truth and estimated point cloud are shown after alignment seen from a bird’s eye view. In Figure 14, the same models are depicted, but this time after they have been projected onto the ground plane to make it easier to evaluate the accuracy. Visually, there are little to no discrepancies noticeable between the two 3D point cloud models.

**Figure 15:**
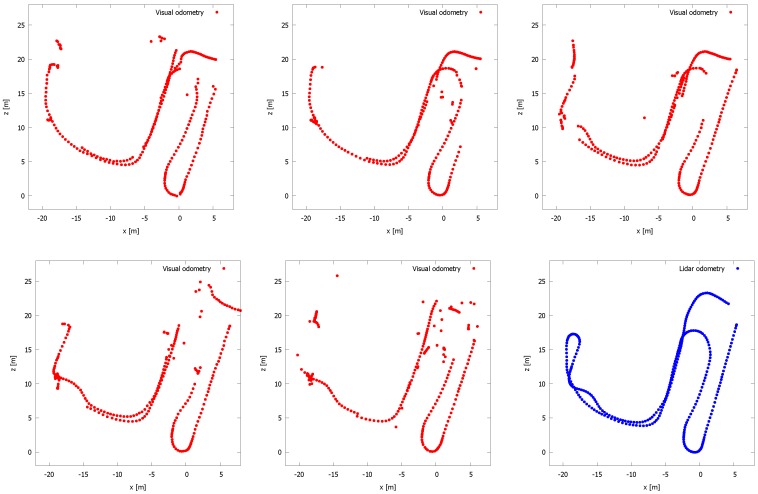
Five plots of the trajectories estimated by the visual structure from motion (SfM) framework presented in [28] using the images of each Ladybug camera separately (red color). The result of the camera pointing upwards is omitted. The blue plot represents the trajectory estimated using our method. For the visual SfM approach, many poses are missing due to the lack of good feature matches. Moreover, many outlier poses are present.

**Figure 16:**
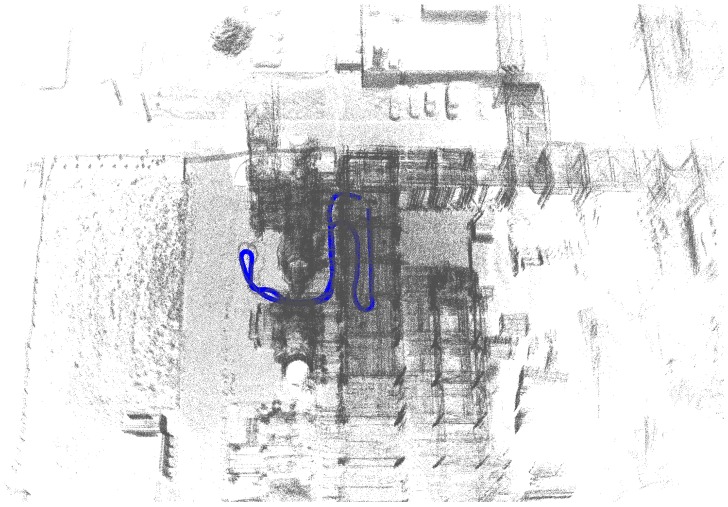
Bird’s eye view of the obtained 3D reconstruction and trajectory of a data sequence captured at the chemical site of Dow company using LiDAR data acquired by the Vellady platform. The blue line is representing the estimated trajectory of the mobile observer.

By means of comparison, the trajectory of the mobile observer is plotted in Figure 15. Herein, six plots are depicted representing the trajectories obtained by the visual structure from motion (SfM) approach using SIFT features, presented in [28] (in red) and the trajectory obtained by our proposed LiDAR mapping system (in blue). Each red graph is the result of the visual SfM method run on the output of one camera of the Ladybug system. As can be clearly seen, many poses are missing for this approach. This is due to the fact that sometimes too few good matches could be found between one image and the others. As a consequence, the resulting point cloud of the visual SfM is far more sparse than the one obtained by the LiDAR system. In addition, many outlier poses are present. The graphs show that the performance of visual SfM is less compared to odometry and mapping using LiDAR data, and hence, the former is less suited to obtain an accurate 3D model of the environment using a mobile observer. Finally, Figure 16 shows an image of the estimated trajectory, as well as the obtained 3D reconstruction using our proposed approach using the LiDAR data acquired by our Vellady platform.

**Figure 17:**
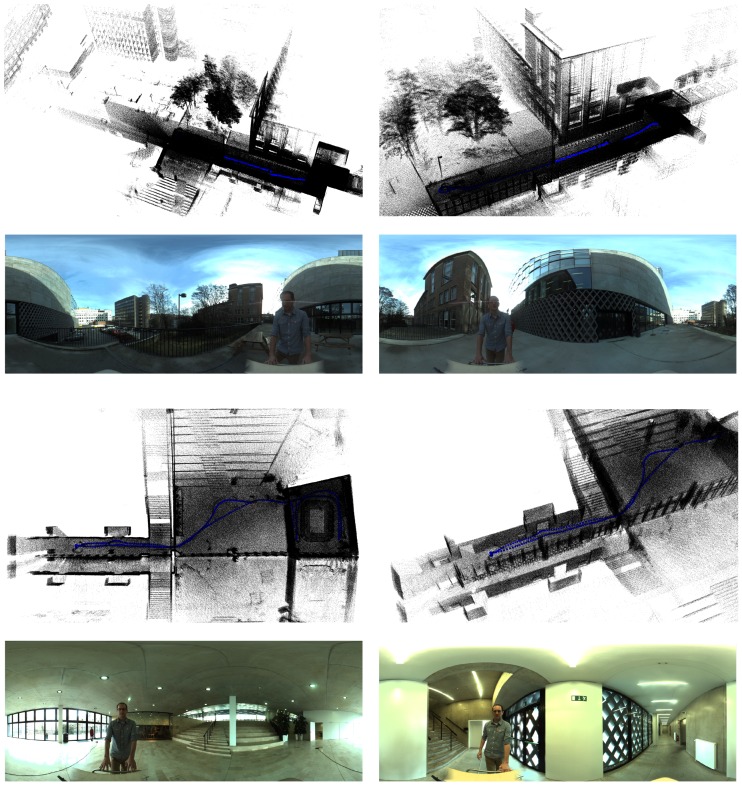
Some example images of the 3D reconstructions obtained by applying our algorithm on the recordings of a campus of Ghent University.

By means of further evaluation, a second set of sequences was captured at a campus of Ghent University. Some example images of 3D reconstructions are shown in Figure 17. Unfortunately, there is no ground truth available for these environments. Visually, we can see that the reconstructions are adequate. The reconstructions of the indoor environment (images at the bottom) however have a mirror effect due to the reflectance of the windows.

### 5.2. Kitti Vision Benchmark

**Figure 18:**
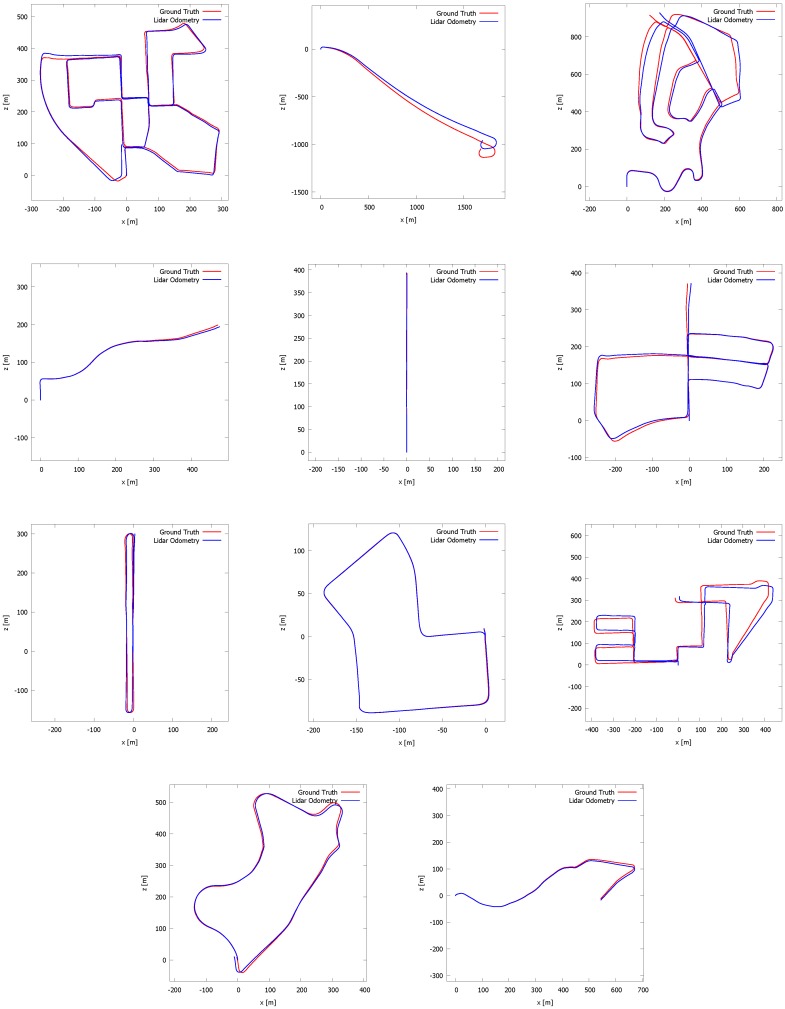
Eleven plots of the estimated (blue) and ground truth (red) trajectories of the Kitti benchmark presented in [27]. The sequences “00” to “10” are presented from left to right and from top to bottom. The algorithm was run without the loop detection and closure algorithm. The majority of the results are satisfying.

To further evaluate our system, we performed experiments on the Kitti vision benchmark presented by A. Geiger in [27], currently one of the main benchmarks related to (visual) odometry. For that benchmark, a van was driving in the streets of the German city Karlsruhe, thereby recording data from different modalities, among them a Velodyne HDL-64e LiDAR scanner and a stereo camera rig. To evaluate our odometry system, we ran our algorithm on the eleven test sequences that were recorded. Important to note is that the sensor set-up by which the sequences were captured differs from our set-up in the sense that the LiDAR scanner was placed perpendicularly. In order to evaluate our results quantitatively, we projected the ground truth and estimated poses on the ground plane as is suggested by the Kitti vision benchmark. The plots of these trajectories are depicted in Figure 18. Visually, the graphs show that our method is capable of approximating the ground truth in most cases. For the very long sequences, the system suffers from drift as is inherent to the SLAM problem. These results were also obtained without the loop detection and loop closure as described in Section 4.6. Next, we derived the translational error $e_{t}$ and rotational error $e_{r}$ from the trajectories. In order to evaluate both errors, the trajectories were quantized in intervals of length Δ. The sensor pose $S_{f}$ corresponding with sweep *f* represents the first sensor pose of the interval, whereas $S_{l}$ represents the last sensor pose of the interval. For $S_{f}$ and $S_{l}$, the following condition is valid: $\left|\right|t_{f}-t_{l}{\left|\right|}_{2}\approx \Delta$. We now define the difference of the two ground truth poses $S_{f}$ and $S_{l}$ and estimated poses ${\hat{S}}_{f}$ and ${\hat{S}}_{l}$ as $S_{\Delta }=S_{f}^{-1}S_{l}$ and ${\hat{S}}_{\Delta }={\hat{S}}_{f}^{-1}{\hat{S}}_{l}$, respectively. The final pose error $E_{\Delta }$ is then given by Equation (14):

(14) $$\begin{array}{cc} E_{\Delta } & =\left[\begin{array}{cc} R_{e} & t_{e}\\ 0_{3}^{⊤} & 1 \end{array}\right]={\hat{S}}_{\Delta }^{-1}S_{\Delta }. \end{array}$$

Finally, the rotational error $e_{r}$ and translational error $e_{t}$ are given by Equations (16) and (17):

(15) $$\begin{array}{cc} d & =\frac{1}{2}\left(\mathrm{tr}\left(R_{e}\right)-1\right), \end{array}$$

(16) $$\begin{array}{cc} e_{r} & =\frac{1}{\Delta }\mathrm{acos}\left(\max\left(\min\left(d,1\right),-1\right)\right), \end{array}$$

(17) $$\begin{array}{cc} e_{t} & =\frac{1}{\Delta }\left|\right|t_{e}{\left|\right|}_{2}. \end{array}$$

**Figure 19:**
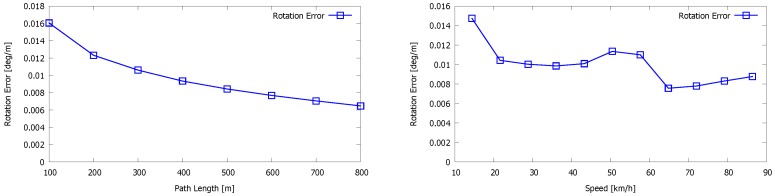
Results of the Kitti vision benchmark showing the average rotation error of all test sequences. Left: the rotation error is expressed as a function of the path length. Right: the rotation error is expressed as a function of the speed.

**Figure 20:**
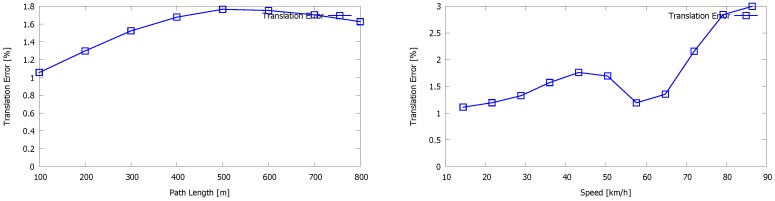
Results of the Kitti vision benchmark showing the average translation error of all test sequences. Left: the translation error is expressed as a function of the path length. Right: the translation error is expressed as a function of the speed.

The graphs that summarize the translational and rotational errors for Δ∈{100,…,800} are depicted in Figure 19 and Figure 20. These graphs are the average numbers for the eleven different sequences of the Kitti dataset. As can be seen, the rotational error decreases from $0.016^{\circ }$ per meter to $0.006^{\circ }$ per meter when the path length Δ increases from 100 to 800 meter. The translational error on the other hand increases from 1% to 1.75% per meter for the path length increasing from 100 to 500. After that, it stabilizes more or less. Regarding the translational error, our algorithm competes with the methods ranked seventh to 20th in the Kitti vision benchmark. Figure 20 also shows the translational and rotational errors for an increasing speed. The rotational error varies only slightly when the speed increases. Regarding the translational error, we can see that it increases for higher speeds, which is expected. For speeds higher than 70 km/h, the error increases drastically. The reason for this failure is clear, as for higher speeds, consecutive point clouds begin to differ greatly from each other and no good correspondences can be found. However, we want to point up that our 3D mapping system is meant to operate in industrial plants or indoor environments where the speed of a mobile observer or a drone is always limited. Our system can perfectly cope with speeds up to 30 km/h.

**Figure 21:**
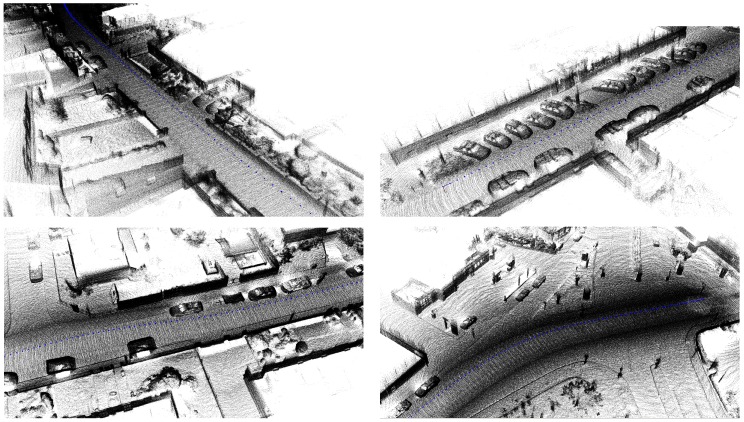
Results of the Kitti vision benchmark showing 3D reconstructions of the validation sequences “11”, “13”, “15” and “18” (left to right, top to bottom) for which no ground truth is provided.

Moreover, our method is more generic, as it does not pose any restriction on the sensor set-up nor any prior knowledge of the type of the scene. Furthermore, it does not rely on the detection of the ground plane, and as a result, it does not need to be present in the LiDAR image. Although the latter assumption could improve the accuracy of the system, we want it to be generic and applicable in any environment and in combination with any sensor set-up. Finally, some example images of 3D reconstructions using the Kitti benchmark are presented in Figure 21. These reconstructions are part of the validation sequences for which no ground truth is provided.

## 6. Conclusions

In this paper, a novel mobile mapping system using a multi-modal sensor set-up was presented. The platform is unique in the sense that it copes with the inhomogeneity of the point clouds produced by LiDAR scanners. To this end, it integrates both an intensive surface analysis, as well as a global map that is continuously updated and improved. Moreover, loop detection is conduction using the output of the Ladybug images, and a loop closure technique was proposed based on the output of the LiDAR odometry. Experiments demonstrated that our system is able to reconstruct challenging environments, such as chemical plants, and can compete with state of the art methods concerning LiDAR mapping in the field of autonomous vehicles; cf. the Kitti vision benchmark.

## Figures and Tables

**Table 1:** A summary containing the main features of the acquisition platform.

	Sensor	Feature
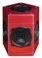	Ladybug system	- 6 camera’s in pentagonal prism, one pointing upwards
		- resolution of 1600 × 1200 per image
		- 1 frame (6 images) per second
		- mounted perpendicular w.r.t. the ground plane
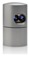	Velodyne HDL-32e	- 360${}^{\circ }$ horizontal FOV, 41.3${}^{\circ }$ vertical FOV
		- 32 lasers spinning at 10 sweeps per second
		- ±700,000 points per sweep
		- mounted with an angle of 66${}^{\circ }$ w.r.t. the ground plane

**Table 2:** The difference in angle between two corresponding planes $\phi \left(H_{s},H_{t}\right)≜\mathrm{acos}\left(n_{s}\cdot n_{t}\right)$ (in degrees) and the average distance of two corresponding planes $d\left(H_{s},H_{t}\right)≜\frac{1}{|H_{t}|}\sum_{p_{t}\in H_{t}}\langle p_{t},n_{s}\rangle -d_{s}$ (in meter) for the eight most dominant planes in the scene.

Plane	$d(\phi_{g},\phi_{r})$	$d_{\mu }\left(H_{g},H_{r}\right)$
1	0.759761	0.00818437
2	0.276246	0.00724192
3	1.12969	0.008299
4	0.286674	0.0115731
5	0.292083	0.0164202
6	0.492578	0.0106246
7	1.80667	0.0118678
8	1.68458	0.00993411
	0.841036	0.0105181
